# Effect of Cellulose Nanocrystals Extracted from Oil Palm Empty Fruit Bunch as Green Admixture for Mortar

**DOI:** 10.1038/s41598-020-63575-7

**Published:** 2020-04-14

**Authors:** Dianah Mazlan, Santhana Krishnan, Mohd Fadhil Md Din, Chiharu Tokoro, Nur Hafizah Abd Khalid, Izni Syahrizal Ibrahim, Hideki Takahashi, Daisuke Komori

**Affiliations:** 1 Centre for Environmental Sustainability and Water Security (IPASA), Research Institute of Sustainable Environment (RISE), School of Civil Engineering, Faculty of Engineering, Universiti Teknologi Malaysia (UTM), 81310 Skudai, Malaysia; 2 Department of Earth Sciences, Resources and Environmental Engineering, School of Creative Science and Engineering, Waseda University, 3-4-1 Okubo, Shinjuku-ku, 169-8555 Tokyo, Japan; 3 Department of Structure and Materials, School of Civil Engineering, Faculty of Engineering, Universiti Teknologi Malaysia, 81310 Skudai, Johor Malaysia; 4 Department of Civil Engineering, Tohoku University, 6-6-06, Aoba, Aramaki, Aoba-ku, 980-8579 Sendai, Japan; 5 Key Laboratory for Green & Advanced Civil Engineering Materials and Application Technology of Hunan Province, College of Civil Engineering, Hunan University, 410082 Changsha, Hunan Province China

**Keywords:** Environmental sciences, Environmental sciences

## Abstract

This paper aims to examine the effect of cellulose nanocrystals (CNCs) derived from oil palm empty fruit bunch fiber (EFB) incorporating cement mortar on its structural performances. Cellulose nanocrystals (CNCs) were extracted from α-cellulose extracted from EFB using an acid hydrolysis process with a concentration of acid used was 64% w/v under the temperature of 45 °C for 60 minutes. The Cellulose nanocrystals (CNCs) were mixed into the cement mortar ranging from 0 to 0.8% w/w and its mechanical properties were determined. The developed CNCs mortar was characterized for their compressive and flexural properties as well as microstructure. The influence of CNCs concentration, curing method, dispersion of CNCs on mortar’s mechanical performance was thoroughly examined to find out the optimum condition. Overall results revealed that an addition of 0.4% cellulose nanocrystals has shown to increase the compressive and flexural strength to 46% and 20%, respectively cured under the wrapping method. The hydration of cementitious composites also improved significantly with the addition of CNCs by the formation of highly crystalline of portlandite observed under the XRD test. This present work demonstrates the importance of palm oil empty fruit bunch waste as a sustainable resource of cellulose nanocrystals admixture to achieve structural strength of cement mortar and promotes green technologies in construction.

## Introduction

Recent times have seen higher demand for sustainable and green products reproduced from waste materials^1^. In particular, this has called for more research and development endeavors to recycle waste materials into biodegradable products with low environmental impacts^2^. The potential of several types of cellulose-based materials from agro-wastes such as palm oil, pineapple, kenaf, sisal, etc. as reinforcing materials in cement composites has been discovered decades ago, but yet to be thoroughly understood and applied in real-world construction^3,4^. Cellulose can be extracted from various plants while oil palm empty fruit bunch is one of its sources. Malaysia is the second-largest producer of palm oil and the country’s palm oil industry produces about 90 million tonnes of lignocellulosic biomass, including empty fruit bunches, oil palm trunks, and oil palm fronds, as well as palm oil mill effluent^5^. Reinforcement of natural fibers and cementitious matrices from various sources of plant fiber have found to improve the mechanical strength of the composites and currently is being applied in various industrial sectors including construction, automobiles, aerospace’s, etc.^6,7^. In particular, renewable waste materials such as agro-waste have enhanced the mechanical properties of cement mortar^8^. However, direct incorporation of natural fiber material into mortar leads to low concrete workability, decay problems, low resistance to chemical attack, and other structural problems^9^. The most common approach to overcome these problems is through surface modification of plant fibers using various chemical treatments (alkali, silane, ozone, etc.) and physical treatment (plasma, UV radiation, corona, etc.)^10–12^. Another approach to improve the properties of natural fibers is through a top-down approach by deconstructing the structure of the plant itself into nano or microfibrous materials such as nanocrystalline cellulose (CNCs) and microcrystalline cellulose (MCC)^13^.

In recent days, construction industries conduct various researches to discover new eco-friendly reinforcement materials to reduce cement usage. Previous few examples of the reinforcement material that contributes to minimizing the cement usage include steel, glass, carbon, etc.^14,15^. In order to replace this material, various high-performance nanostructures such as carbon nanotubes have been utilized to improve the performance of cement composites^16,17^. However, troubles in dispersion and cost-effectiveness are the major crisis with these materials, which are needed to be addressed before commercialization^7^. At present, the performance of nano and micro cellulose materials as reinforcement of cementitious composites is getting research attention worldwide. Positive result discovered by Cao *et al*.^12^ through the use of CNCs extracted by using the acid hydrolysis process. An enhancement of cement mortar up to 30% for the flexural strength with the addition of 0.2% CNCs was found^12^. Another achievement found with the addition of CNCs in oil well cement studied by Reza *et al*.^18^, revealed that CNCs reduced the porosity up to 33%, surface area to 66% and 0.7% of the total design water by mass. Also, CNCs have raised the compressive and tensile strength by 60% in the first 24 hours of composite’s age.

However, research on CNCs based cementitious composites extracted from palm oil fruit bunches are very rare in the existing literature. Therefore, this present study intends to report the effect of CNCs suspension on the mechanical properties of mortar via different: i) curing environment, ii) percentage of CNCs added and iii) morphological observation. A series of experiments were conducted to examine the effect of different curing methods (water, lime and wrapping curing) to find the best method of curing, as well as the microstructural and mechanical properties of the cement composites after adding CNCs.

## Materials and Methods

The cement mortar mix used in this study were prepared by mixing the CNCs aqueous suspension together with fine aggregates and cement at 0.5 water/cement ratio^19^. The amount of CNCs liquid suspension added to cement composites was from 0% to 0.8% by volume of cement content. The dispersion behavior of the CNCs in aqueous suspension was found to be more stable compared to the powder form.

### Preparation of the CNCs

The cellulose was supplied by a local company from Waris Nove Sdn. Bhd. at Kuantan, Pahang Malaysia that commercially produces α-cellulose from palm oil wastes for industrial applications. The extraction process began by referring to the extraction methods from Dong *et al*.^20^ and Lu and Hsieh^21^ with the production of CNCs from α-cellulose. The first step is to extract the microcrystalline cellulose (MCCs) from α-cellulose^20^. In order to produce MCC, 2.5 N hydrochloric acid (HCl) was mixed with α-cellulose and incubated for 15 minutes at the controlled temperature of 105 °C. This is followed by the addition of cold water to the hot mixture, stirred and the mixture was left overnight. The mixture was filtered and then washed with water until the pH reached 6~7. The filtered sample was dried in a hot air oven for 60 minutes at 60 °C. The finally the sample was ground and sieved using 60 µm sieve aperture before it was extracted for CNCs.

Based on Kumar *et al*.^22^, during the CNCs extraction, 64% w/v sulphuric acid (H_2_SO_4_) was used for the acid hydrolysis process. The acid solution was initially preheated to 45 °C and MCCs were added at a ratio of 10:1 (diluted H_2_SO_4_: MCCs). Subsequently, the solution was stirred for 60 minutes, mixed with 1/10 fold of chilled distilled water, and centrifuged at 6000 rpm for 15 minutes to remove any excessive acid. Then, the remaining precipitate underwent dialysis for 5-7 days for neutralization. Finally, the solution was centrifuged and subjected to sonification for 15 minutes to form CNCs aggregates. The final product was refrigerated at 4 °C until the further application.

**Figure 1:**
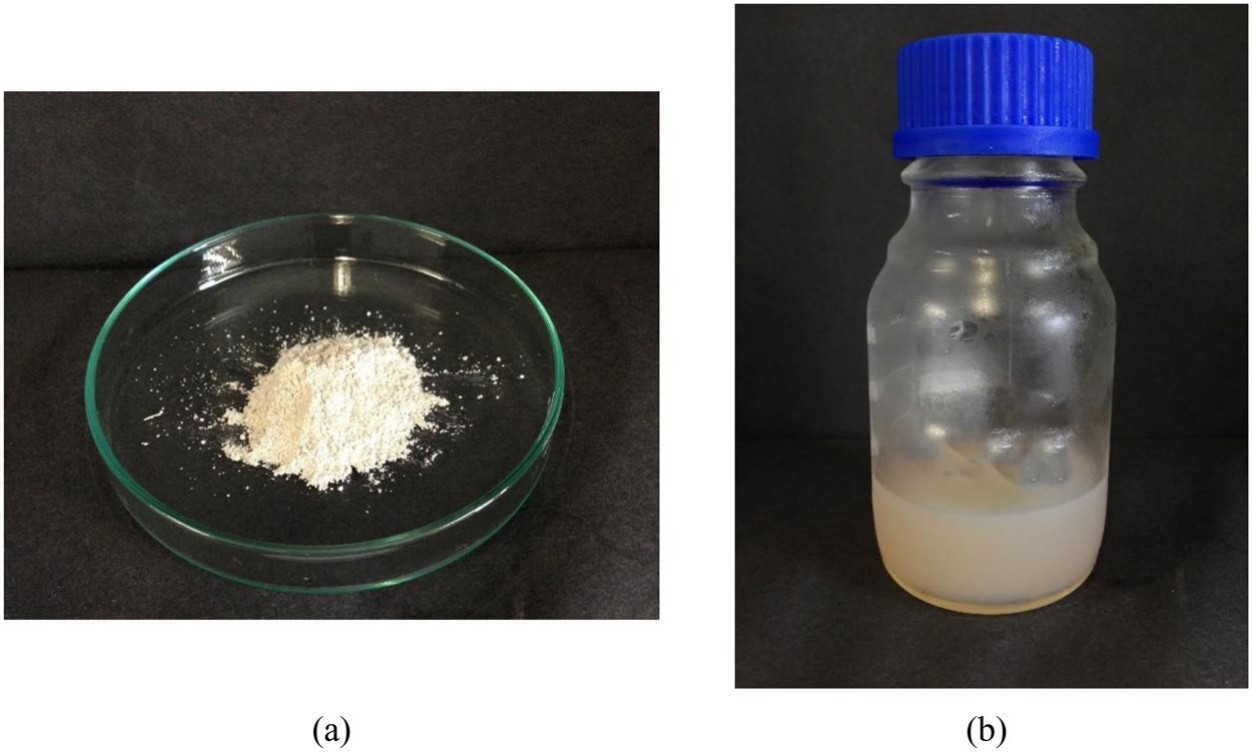
Two different types of cellulose production (**a**) MCC powder (**b**) CNCs aqueous suspension.

**Figure 2:**
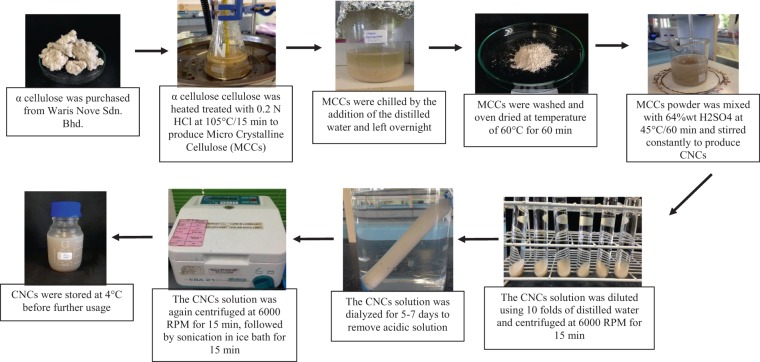
Schematic illustration of CNCs extraction process.

In general, when MCCs was mixed with sulphuric acid (through acid hydrolysis process) it changes the MCCs to CNCs^23–25^. Sulfuric acid hydrolysis of cellulose is a heterogeneous process where the acid diffuses into the pulp fiber and cleaves the glycosidic bonds in the cellulose polymer. Depending on reaction times, temperature, and how the heating rate is controlled, the hydrolysis could also occur on the crystalline regions and some of the hydroxyl groups on the crystalline surface and convert into sulfate groups (e.g., conversion of cellulose-OH to cellulose-OSO_3_−H^+^). CNCs can be generated which is in a milky white color but not as brownish or blackish color. The brownish and blackish color solution shows that the CNC is burned due to improper selection of acid concentration and temperature. Therefore, in this study concentration of sulphuric acid used was 64% w/v with a temperature of 45 °C for 60 minutes. With this condition of acid hydrolysis, the end product of CNC comes out to be milky white in color. Figure 1(a) shows the MCCs powder after the α-cellulose pre-treatment process and Fig. 1(b) displays the CNCs aqueous suspension after the acid hydrolysis process (milky white in color). The schematic illustration of the CNCs extraction process was simplified in Fig. 2. The chemical composition of the extracted CNCs from palm oil wastes used in this study was evaluated via X-ray Fluorescence (XRF) assessment and tabulated in Table 1. The main constituents of the CNCs were detected as carbon and oxygen. A low amount of sulfur was also detected, most probably from the leftover acid during the acid hydrolysis process with the H_2_SO_4_ solution.

**Table 1:** Chemical composition of cellulose nanocrystals used as an admixture in cement composites.

Chemical Composition	Mass Percentage (%)
Carbon, C	43.76
Oxygen, O	55.12
Sulfur, S	1.01
Others	0.11

### Cement mortar samples preparation and strength tests

#### Cement and sand preparation

Portland Cement Type I was used throughout the study. This was obtained from a Tasek Cement Company located at Ipoh, Malaysia. Based on ASTM C778-113^26^, two types of sand with different sizes were blended in the equal portion into the mortar mixes, i.e passing 850-µm sieve and retained at 600-µm sieve and passing 600-µm sieve and retained at 150-µm sieve^26^.

#### Mortar samples casting

The specimens consisted of 50 mm cubes and 40 mm by 40 mm by 160 mm prism for compressive and flexural strength tests, respectively. By following the ASTM C109/C109M^19^, the specimens were prepared based on the optimum mix design of 1:2.75 (cement:sand) with a water/cement (w/c) ratio of 0.5 and five different content of CNCs by volume of cement (0% for control specimen, increased every 0.2% until reaching 0.8%) as additive. Based on previous literature, the addition of CNCs in cement composites only up to 0.2% and 0.4%. Therefore, a range of 0% (as a control specimen) to 0.8% is chosen in this study to understand the effect of CNCs addition in mortar can improve or reduce its performance in strength^27–29^. The workability of the mortar was obtained via the flow table method as stated in ASTM C230/C230M-14^30^. After mixing, the mortar was poured into the specimen moulds and compacted using a vibrating table for two minutes with a vibrating rate of 12000 ± 400/minute^30^.

#### Mortar samples curing procedures

The hardened mortar specimen was cured in three different conditions – water, lime, and wrap curing for 28 days. Water curing was done by immersing the mortar samples into tap water until it reaches the testing day. As for lime curing, the specimens were prepared using a saturated lime solution, which was prepared by mixing 2 grams of hydrated lime in every liter of water. Wrap curing was conducted with at least three layers of polyethylene film wrapping onto mortar samples to prevent water from evaporating during the hydration process. The ambient temperature was maintained at 23 ± 2 °C with more than 50% humidity. Finally, all samples were tested for compressive strength. All the experiments were performed in triplicates.

#### Mechanical properties

The mechanical properties of the cement composite with CNCs were studied for its compressive and flexural strength for 7, 14 and 28 days. Compressive and flexural strength test was evaluated using a Universal Testing Machine (MATEST3000) with a maximum loading capacity of 3000 kN and a pacing rate of 0.9 kN/s for compressive strength and 0.04 kN/s for testing flexural strength test^31^.

### CNCs-mortar characterization

The microstructure of the CNCs mortar was studied under Field Emission Scanning Electron Microscope (FESEM, JSM 670-1 F, JEOL, Japan) and Scanning Electron Microscope/Energy Dispersive X-ray Spectroscopy (SEM/EDS, Quanta 650 MLA-FEG, FEI, Australia, acceleration voltage: 20 kV, coating:30 nm Au-Pd) to understand the changes induced by CNCs on cement composites. Since the resolution of SEM/EDS observation has its limitations, the FESEM test was conducted to further observe morphological changes previously identified by the SEM/EDS under the higher resolution. During this morphological study, various magnifications were applied to the control sample and CNCs-mortar sample. For instance, using FESEM, the magnification was 25,000 times for the control sample, but 10,000 times for the CNCs-mortar sample. The same applies to the SEM/EDS monitoring, with magnification was set at 766 times for the control sample and 854 times for CNCs-mortar sample.

In order to study the crystalline pattern of calcium hydroxide formation (portlandite) in cement composites after incorporating CNCs, the X-ray Diffraction (XRD, Rint 2000 Ultima- III, RIGAKU Corporation, Japan) test was conducted using an X-ray Diffractometer with graphite filtered CuK (λ = 1.5433 Å) radiation at 40 kV and 30 mA. The data were collected on a 2θ scale from 0 to 80°.

The zeta potential evaluation was done by Zetasizer Nano ZS90: DLS Zeta Potential Analyzer from Malvern Instrument for cement particle and CNCs aqueous suspension to study the dispersion behavior of CNCs before and after mixing with the cement materials. This is important to make sure the CNCs can be uniformly distributed instead of becoming agglomerated during the mixing process. For this reason, both samples were diluted with distilled water until achieving a concentration of 0.01 mol/l with pH 7. The refractive index of the CNCs and cement particles were set to 1.46 and 1.35, respectively.

## Results and Discussion

### The effects of different curing methods on CNCs mortar

**Figure 3:**
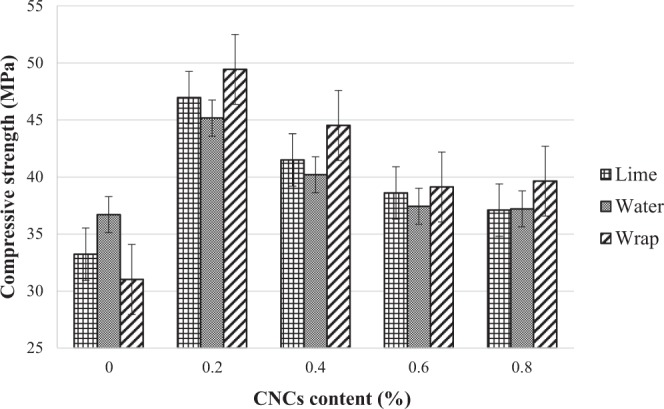
Compressive strength of cement mortar at age of 28 days with different curing methods and CNCs concentration.

As depicted in Fig. 3, all operational parameters of different curing methods have resulted in a myriad of strengths. Among all curing methods, the wrapping method showed the highest improvement in strength after 0.2% of CNCs were added into the mortar matrix, achieving a compressive strength of 49 MPa at the age of 28 days with a 48% increment compared to the control mortar. On the other hand, for lime and water curing methods, the compressive strength of 47 MPa and 43 MPa, respectively, was recorded with the same amount of CNCs added; these are translated into 43% and 42% increment, respectively, compared to the control sample. Therefore, the wrap method was chosen to be used as the curing method throughout the study due to its effect on compressive strength.

The hydration of cement mortar must have adequate control of moisture and temperature movement from and into the composites’ matrices. Furthermore, a proper curing process -ensures good protection of cement mortar against damage due to loading and mechanical interference^2,32^. Fundamentally, the resistance towards loading and mechanical impacts comes from a good production of calcium crystals (calcium-silicate-hydrate, C-S-H, and calcium hydroxide, Ca(OH)_2_) compounds in cement mortar^33^. These compounds form after the water combines with cement particle compounds (tricalcium silicate, C_3_S), as stated in Eq. (1) below:

1 $${{\rm{C}}}_{3}{\rm{S}}+(1.3+{\rm{x}}){\rm{H}}\to {{\rm{C}}}_{17}{{\rm{SH}}}_{{\rm{x}}}+1.3{\rm{CH}}.$$

Where:

C_3_S = tricalcium silicate

CSH = calcium silicate hydrate

CH = calcium hydroxide

Equation (1) was developed by Allen *et al*.^34^ where “x” denotes the variables of water. This shows that the amount of water available during the curing process greatly affects the production of calcium crystals during hydration. On the contrary, insufficient moisture promotes the formation of pores and microcapillary pores, which directly decreases the strength and long-term durability of the concrete.

The lime and water curing methods stated in this study have undoubtedly provided sufficient moisture for hydration compared to the wrapping method. However, the wrapping method had unexpectedly given the best result in terms of strength. This is attributed to the ability of moisture preservation (vapor pressure) for optimum hydration. Hence, by controlling the movement of water outside and inside the cement matrices during the curing process, a cement composite with higher resistance to loading impact can be produced^35–37^.

The different compressive strength recorded for different curing methods is also attributed to the addition of CNCs into the matrices. CNCs are known as cellulose-based nanofibers which are built up of hydrophilic nanoporous structure. The expansion and shrinkage of this structure during and after the curing period might have led to microcracking, and consequently, a lower strength than the designed strength. With an obvious lower strength recorded for water and lime curing methods, it becomes clear that cement mortar containing cellulose-based materials such as CNCs ought to be cured using unconventional methods. Zheng *et al*.^32^ and Mariano *et al*.^33^. have also stated this, where the authors affirmed that the hydrophilic nature of CNCs which causes the structure to absorb and store water is profound and should not be ignored. As such, its curing process should be investigated and studied in-depth.

### Mechanical properties

#### Effect of CNCs on cement mortar strength

**Figure 4:**
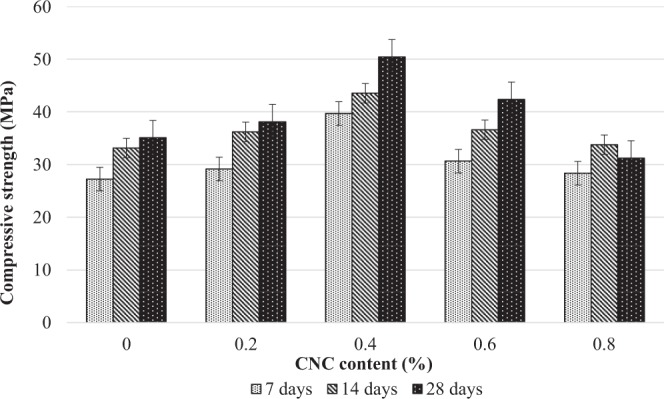
Compressive strength of cement mortar incorporating different percentage of CNCs concentrations under wrap curing condition.

The compressive strength performance of cement mortar with CNCs cured under wrapping technique is graphically represented in Fig. 4. With the addition of 0.4% CNCs, the compressive strength increased between 43% and 46% compared to the control sample after 28 days of curing period. Further addition of CNCs did not show a significant increase in strength. For instance, the addition of 0.6% and 0.8%, CNCs showed the compressive strength up to 16% and 38%, respectively. Comparison of previous reports in Table 2 shows that CNCs have a high potential in improving the strength of cement composites after its addition. In this study, the optimum CNCs content was found at 0.4%. This was due to the reason that the CNCs had changed the structural properties of the cement mortar. An increase in strength could mean that the CNCs had triggered a good production of C-S-H gel and Ca(OH)_2_ during the hydration process, which is an important component for strength development other than Ca(OH)_2_^12^. The function of these hydration products is to bind the cement particles together^38^. Though this may be interpreted as the more the binder, the stronger the structure could be, it is apparent that a CNCs content of more than 0.4% does not cause any changes in strength compared to the conventional mortar. This is because the high amount of hydration products formed could have made the structure more brittle and thus, more prone to cracking^39^.

**Table 2:** Comparison of previous reports on the effect of CNCs addition in to mortar.

Parameters	Findings	Literature	Reflection
Extraction method	-Cellulose source: Palm oil empty fruit bunch	[1] Fortunati *et al*., (2013)^52^ -Cellulose source: Okra bahmia -Process: Acid hydrolysis -Condition: 64 wt/wt % H_2_SO_4_ at 45 °C for 30 min	Different raw sources of cellulose required different acid hydrolysis condition to extract the CNCs
	-Process: Acid hydrolysis	[2] Nuruddin *et al*., (2014)^53^ -Cellulose source: Kenaf fiber -Process: Acid hydrolysis -Condition :60% H_2_SO_4_ (v/v) at 50 °C for 60 min	
	-Condition: 64% w/v H_2_SO_4_ at 45 °C for 60 min	[3] Darpentigny *et al*., (2019)^54^ -Cellulose source: Tunicate -Process: Acid hydrolysis -Condition: 50 wt% H_2_SO_4_ at 50 °C for 20 hours	
Compressive strength	Improved by 43% to 46% compared to conventional mortar	[1] Barnat-Hunek (2019)^55^ found that with 1.5% addition of CNCs improved 27.6% of compressive strength	The addition of CNC in cement composites improved the compressive strength performance of more than 20% of the cement composite’s original strength. Compressive strength of cement composites
		[2] Aloulou *et al*., (2019)^56^ proved that compressive strength improved by 50% with the addition of 1% nano wood fiber.	
		[3] As mentioned by Jiao *et al*., (2016)^57^, 0.15% addition of CNCs will improve the compressive strength of cement by 20%	
Flexural strength	Improved by 20% compared to conventional mortar	[1] Barnat-Hunek (2019)^55^ mentioned that with the addition of 1.5%CNCs improved flexural strength of mortar by 10.9%	CNCs have a high potential in improving cement composite flexural strength more than
		[2] Fu *et al*., (2017)^58^ reported that flexural strength improved by 20% with the addition of 0.2% of CNCs	10% of normal cement composites strength.
		[3] Jiao *et al*., (2016)^57^ presented the data of flexural strength improvement by 15% after the addition of 0.15% CNCs in cement paste.	
		[4] Cao *et al*., (2013)^29^ found that with 0.2% CNCs improve flexural strength by 30%	

**Figure 5:**
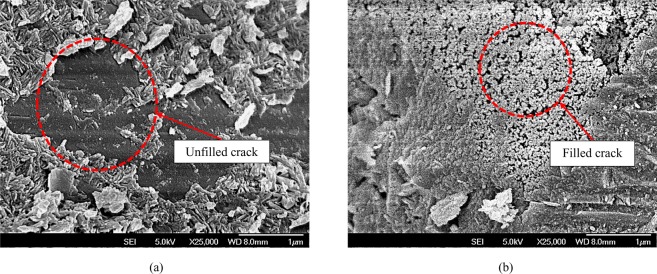
FESEM image of calcium crystals (C-S-H and Ca(OH)_2_) filled in mortar specimen (**a**) control (**b**) 0.2% CNCs.

With this, morphological studies were conducted to further understand the effect of adding CNCs into cement mortar using FESEM and SEM/EDX plus XRD characterization. Figure 5 shows the FESEM results between conventional mortar (Fig. 5a) and mortar added with CNCs (Fig. 5b). Apparently, the formation of hydration products was more obvious in CNCs-mortar. The C-S-H gel and Ca(OH)_2_ compounds had also filled in the pores, making the mortar structure denser and more compacted; the CNCs had not only functioned as nuclei to induce nucleation of hydration products but also a bio base nano-filler^40^. The second function has been extensively discussed by Maraino *et al*.^41^, where it is stated that its unique and favorable nano-characteristics include large surface area and aspect ratio.

**Figure 6:**
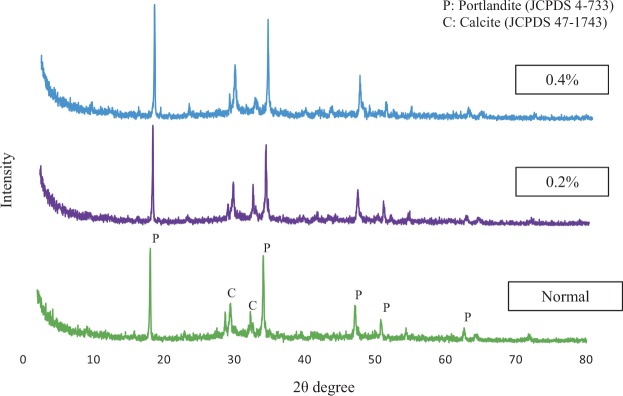
XRD pattern of control sample and 0.2% CNCs-mortar at 28 days. P: Portlandite (Ca(OH)_2_), CS: Calcium-Silicate-Hydrate (C-S-H), Et: Ettringite.

The XRD data were used to further confirm the existence of hydration products in addition to FESEM images (see Fig. 6). The highest peaks at 18, 34, 47 and 51 denoted the existence of calcium hydroxide, Ca(OH)_2_, also known by its mineral name portlandite, was found higher in the mortar-CNCs sample than the control^42^. Calcium hydroxide forms as crystals with a wide range of shapes and sizes, which can completely engulf a small cement particle next to it^43^. A significant proportion of the calcium hydroxide formed as an intimate mixture with the C-S-H gel. C-S-H gel tends to be much smaller in which their growth could have impeded by the surrounding solid^44^. Therefore, it’s hard to distinguish since the latter contains a significant proportion of Ca-OH bonds. Since, the strength of cement mortar depends on the production of C-S-H gel, more formation of C-S-H gel, results in better strength of cement mortar. However, from the XRD test the C-S-H formation is hard to measure correctly due to its amorphous behavior and poorly crystalline. Therefore, the crystallinity of portlandite (Ca(OH)_2_) was measure instead. This is because. the portlandite is a secondary product of hydration (Eq. 1) and its highly crystalline^45^. C-S-H size has nanometer level morphology and the observation of peak in Fig. 6 confirms the presence of portlandite formation as a binding agent which in turn contributes to the higher mechanical strength of cement mortar.

Since the CNCs added in the cement mortar was in aqueous suspension form, the zeta potential of the solution was also determined to affirm its dispersion characteristic during the mixing process as well as its effects, such as whether the CNCs particles had bonded with itself or cement particles. Table 3 shows the zeta potential value of cement and CNCs. The zeta potential of the CNCs aqueous suspension (−50.4 mV), CNCs powder form (−36.1 mV) was much higher than cement particles, which means that it does not agglomerate easily by itself due to the high degree of electrostatic repulsion. This characteristic has arisen due to the earlier acid hydrolysis process which has promoted the formation of sulfate group^46^. The lower zeta potential value of −16.6 mV for cement particles indicated easy agglomeration and the significant difference of zeta potential value between CNCs and cement particles, which attract it to stick together due to make the attractive force exceed the repulsion force. The CNCs particles are more likely to stick to the cement particles since it has higher zeta potential^47^.

**Table 3:** Different zeta potential value between cement particle and cellulose nanocrystals.

Types of particle	Zeta Potential (mV)
Cement	−16.6
CNCs	−50.4

**Figure 7:**
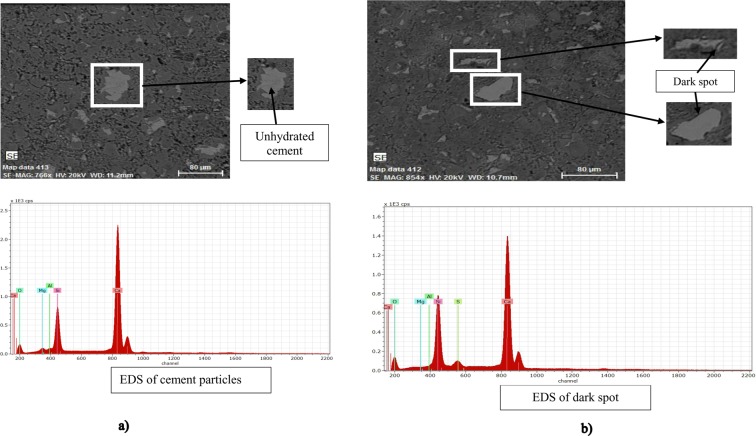
SEM/EDS observation to show the (**a**) unhydrated cement particle of conventional mortar (**b**) CNCs-mortar with dark spot formation around unhydrated cement particles.

Figure 6 reported the SEM/EDS data of the unhydrated cement particles for control mortar (Fig. 7a) and the adhering CNCs around the cement particles for CNCs-mortar (Fig. 7b). Different magnification was used to observe the dark spot around the unhydrated compound. The dark spot occurred only in the sample containing CNCs. The EDS result of the dark spot confirmed the presence of Sulphur (S) as the CNCs contained some sulfate groups (Table 1) that is formed during the acid hydrolysis process. It is expected that CNCs agglomerate around the unhydrated cement particle. However, a dark spot from EDS is difficult to be determined accurately at this point because the CNCs are known to contain light chemical compounds^29^. As preventive measures, 30 spots of EDS pointed at the dark spot and the finding is almost similar to the presence of Sulphur. In order to support the idea of the dark spot identity, the result of X-ray Fluoresces (XRF) of cement particle is given in Table 4. The EDS data of unhydrated cement particles illustrated in Fig. 6a shows the absence of Sulphur content.

**Table 4:** Chemical composition of cement by XRF analysis.

Component	Percentage by weight (%)
Silica, SiO_2_	19.8
Alumina, Al_2_O_3_	5.6
Iron oxide, Fe_2_O_3_	3.4
Calcium oxide, CaO	62.7
Magnesium oxide, MgO	1.2
Sodium oxide, Na_2_O	0.02
Phosphorus pentoxide, P_2_O_5_	0.1
Loss of Ignition, LOI	2.1
Lime saturated factor	1.0

The black or darker spot around the cement particle was found to be the CNCs nanofiber sticking to the cement particles. The dark spot was another reason behind the high formation of calcium crystals when CNCs were added where its hydrophilic behavior had caused absorption of water into the porous structure. The inference made here is that the absorbed water had been the major water supply for the unhydrated cement particles to go through the hydration process and form extra calcium crystals even after the curing period. A similar finding has been described by Cao *et al*.^12^, where the identical dark ring was found around the cement particle in the cement matrix, which was later identified as concentrated CNCs lingering around the cement particles. The finding shows that CNCs can potentially provide continuous strength development in aging cement mortar^48^.

#### Flexural strength performance effected by the addition of CNCs

**Figure 8:**
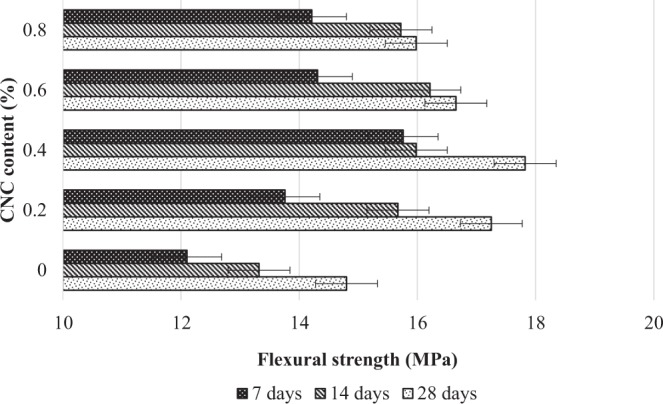
The effect of cement composites containing CNCs as nano reinforced agent.

The performance of flexural strength shows a substantial improvement after the addition of CNCs into the mortar matrix (see Fig. 8). Based on that, the optimum limit of CNCs was 0.4% where an increase in flexural strength of up to 20% stronger than conventional mortar was recorded. However, further addition of CNCs in mortar had resulted in a reduction of 7% to 10% in flexural strength.

The optimum CNCs content for flexural strength was different from the results of compressive strength because of the CNCs could function better in resisting flexural failure when subjected to axial loading^42^. This attribute has yet to be discussed extensively in most of the studies - though the same behavior has been found in many works using carbon nanotubes as the flexural strength booster. These two materials share almost the same nanofiber shape, and thus, theoretically, both can act as the bridging agent or reinforcement material^8^. Parveen *et al*.,^49^ reported that nanomaterials can be effective in preventing the initiation of crack growth in nanoscale level. Without CNCs, cement composites are vulnerable to freeze and thaw damage, chloride penetration, corrosion and alkali-silica reaction, which degrade the overall performance of composites^50^. This occurs when the interfacial interaction between the nanofiber and the bonding agents of the hydration products (C-S-H and Ca(OH)_2_) has enhanced the load transfer between the matrix and reinforcement. Similar findings reported by Nochaiya and Chaipanich^51^, shows that the addition of 0.5% carbon nanotube cause the pore reduction left between C-S-H and Ca(OH)_2_ due to the presence of carbon nanotubes as a filler and bridging agents of hydrated cement.

## Conclusion

The present work concludes the significant impact of CNCs on cement mortar which the CNCs have affected the curing performance of the cement mortar. Wrap curing with polyethylene film was the most effective method, evident through the highest compressive strength recorded. Moreover, the compressive strength of the cement mortar increased 43% to 46% from its original strength when 0.4% of CNCs were added. In addition, the effect of CNCs addition also positively affected the flexural strength of cement mortar that increased about 20% when up to 0.4% CNCs were added. Finally, the addition of CNCs found to change the inner structure of the composites where the formation of calcium crystals (C-S-H or Ca(OH)_2_) that would strengthen the structure continued even after the curing process ended. Further studies on the other potentials of CNCs in improving cement mortar should be examined thoroughly due to its promising characteristics and the possibility of being applied in real-world construction projects.

### Novelty statement

This study is reported to be the novel study on examining the effect of cellulose nanocrystals (CNCs) derived from palm oil empty fruit bunch (EFB) fiber into cement mortar as a green admixture to increase its structural performance.
